# Prospective cohort study of early biosignatures of response to lithium in bipolar-I-disorders: overview of the H2020-funded R-LiNK initiative

**DOI:** 10.1186/s40345-019-0156-x

**Published:** 2019-09-25

**Authors:** Jan Scott, Diego Hidalgo-Mazzei, Rebecca Strawbridge, Allan Young, Matthieu Resche-Rigon, Bruno Etain, Ole A. Andreassen, Michael Bauer, Djamila Bennabi, Andrew M. Blamire, Fawzi Boumezbeur, Paolo Brambilla, Nadia Cattane, Annamaria Cattaneo, Marie Chupin, Klara Coello, Yann Cointepas, Francesc Colom, David A. Cousins, Caroline Dubertret, Edouard Duchesnay, Adele Ferro, Aitana Garcia-Estela, Jose Goikolea, Antoine Grigis, Emmanuel Haffen, Margrethe C. Høegh, Petter Jakobsen, Janos L. Kalman, Lars V. Kessing, Farah Klohn-Saghatolislam, Trine V. Lagerberg, Mikael Landén, Ute Lewitzka, Ashley Lutticke, Nicolas Mazer, Monica Mazzelli, Cristina Mora, Thorsten Muller, Estanislao Mur-Mila, Ketil Joachim Oedegaard, Leif Oltedal, Erik Pålsson, Dimitri Papadopoulos Orfanos, Sergi Papiol, Victor Perez-Sola, Andreas Reif, Philipp Ritter, Roberto Rossi, Thomas Schulze, Fanny Senner, Fiona E. Smith, Letizia Squarcina, Nils Eiel Steen, Pete E. Thelwall, Cristina Varo, Eduard Vieta, Maj Vinberg, Michele Wessa, Lars T. Westlye, Frank Bellivier

**Affiliations:** 1 Institute of Neuroscience, Newcastle University, Newcastle upon Tyne, UK; 2 Centre for Affective Disorders, Department of Psychological Medicine, Institute of Psychiatry, Psychology & Neuroscience, King’s College London, London, UK; 3 Université Paris Diderot, 75013 Paris, France; 4 Bipolar and Depressive Disorders Unit, Department of Psychiatry and Psychology, Institute of Neurosciences, Hospital Clinic de Barcelona, University of Barcelona, IDIBAPS, CIBERSAM, Villaroel 170, 08036 Barcelona, Catalonia Spain; 5 Service de Biostatistique et Information Médicale, Hôpital Saint-Louis, AP-HP, Paris, France; 6 Inserm, UMR 1153, Equipe ECSTRA, Paris, France; 7 Département de Psychiatrie et de Médecine Addictologique, AP-HP, GH Saint-Louis – Lariboisière – F. Widal, 75475 Paris, France; 8 Inserm, U1144, Team 1, 75006 Paris, France; 9 NORMENT Centre, Division of Mental Health and Addiction, Oslo University Hospital, Oslo, Norway; 10 Institute of Clinical Medicine, University of Oslo, Oslo, Norway; 11 Department of Psychiatry and Psychotherapy, University Hospital Carl Gustav Carus, Technische Universität Dresden, Dresden, Germany; 12 Department of Clinical Psychiatry, Inserm CIC 1431, CHU Besançon, 25000 Besançon, France; 13 Laboratoire de Neurosciences, Université Bourgogne Franche-Comté, 25000 Besançon, France; 14 Institute of Cellular Medicine, Newcastle University, Newcastle upon Tyne, NE1 7RU UK; 15 Newcastle Magnetic Resonance Centre, Campus for Ageing and Vitality, Newcastle University, Newcastle upon Tyne, NE4 5PL UK; 16 NeuroSpin, CEA, Université Paris-Saclay, 91191 Gif-sur-Yvette, France; 17 Department of Neurosciences and Mental Health, Fondazione IRCCS Ca’ Granda Ospedale Maggiore Policlinico, University of Milan, Milan, Italy; 18 Department of Psychiatry and Behavioural Neurosciences, University of Texas at Houston, Houston, TX USA; 19 IRCCS Istituto Centro San Giovanni di Dio - Fatebenefratelli, Brescia, Italy; 20 CATI Neuroimaging Platform, ICM, Pitié Salpétrière Hospital, 75013 Paris, France; 21 Institut du Cerveau et de la Moelle épinière, ICM, 75013 Paris, France; 22 Inserm, U1127, 75013 Paris, France; 23 CNRS, UMR 7225, 75013 Paris, France; 24 Sorbonne Université, 75013 Paris, France; 25 Copenhagen Affective Disorder Research Center (CADIC), Psychiatric Center Copenhagen, University Hospital of Copenhagen, Copenhagen, Denmark; 26 Mental Health Research Program, IMIM, Hospital del Mar, CIBERSAM, Barcelona, Catalonia Spain; 27 Northumberland Tyne and Wear NHS Foundation Trust, Newcastle upon Tyne, NE3 3XT UK; 28 APHP; Psychiatry Department, University Hospital Louis Mourier, Colombes, France; 29 INSERM U894, Institute of Psychiatry and Neurosciences of Paris, Paris, France; 30 NORMENT, Division of Psychiatry, Haukeland University Hospital, Bergen, Norway; 31 Department of Clinical Medicine, University of Bergen, Bergen, Norway; 32 Institute of Psychiatric Phenomics and Genomics (IPPG), University Hospital, LMU Munich, Munich, Germany; 33 Department of Psychiatry and Psychotherapy, University Hospital, LMU Munich, Munich, Germany; 34 International Max Planck Research School for Translational Psychiatry (IMPRS-TP), Munich, Germany; 35 Institute of Neuroscience and Physiology, Department of Psychiatry and Neurochemistry, The Sahlgrenska Academy, University of Gothenburg, Gothenburg, Sweden; 36 Mohn Medical Imaging and Visualization Centre, Department of Radiology, Haukeland University Hospital, Bergen, Norway; 37 Department of Psychiatry, Psychosomatic Medicine and Psychotherapy, University Hospital Frankfurt, Frankfurt am Main, Germany; 38 Unit of Psychiatry, IRCCS Istituto Centro San Giovanni di Dio - Fatebenefratelli, Brescia, Italy; 39 Department of Clinical Psychology and Neuropsychology, Institute for Psychology, Johannes Gutenberg-University Mainz, Wallstraße 3, 55122 Mainz, Germany; 40 Department of Psychology, University of Oslo, Oslo, Norway

**Keywords:** Bipolar, Precision, Personalization, Lithium, Response, Phenotype, Digital, Actigraphy, Omics, Neuroimaging

## Abstract

**Background:**

Lithium is recommended as a first line treatment for bipolar disorders. However, only 30% of patients show an optimal outcome and variability in lithium response and tolerability is poorly understood. It remains difficult for clinicians to reliably predict which patients will benefit without recourse to a lengthy treatment trial. Greater precision in the early identification of individuals who are likely to respond to lithium is a significant unmet clinical need.

**Structure:**

The H2020-funded Response to Lithium Network (R-LiNK; http://www.r-link.eu.com/) will undertake a prospective cohort study of over 300 individuals with bipolar-I-disorder who have agreed to commence a trial of lithium treatment following a recommendation by their treating clinician. The study aims to examine the early prediction of lithium response, non-response and tolerability by combining systematic clinical syndrome subtyping with examination of multi-modal biomarkers (or biosignatures), including omics, neuroimaging, and actigraphy, etc. Individuals will be followed up for 24 months and an independent panel will assess and classify each participants’ response to lithium according to predefined criteria that consider evidence of relapse, recurrence, remission, changes in illness activity or treatment failure (e.g. stopping lithium; new prescriptions of other mood stabilizers) and exposure to lithium. Novel elements of this study include the recruitment of a large, multinational, clinically representative sample specifically for the purpose of studying candidate biomarkers and biosignatures; the application of lithium-7 magnetic resonance imaging to explore the distribution of lithium in the brain; development of a digital phenotype (using actigraphy and ecological momentary assessment) to monitor daily variability in symptoms; and economic modelling of the cost-effectiveness of introducing biomarker tests for the customisation of lithium treatment into clinical practice. Also, study participants with sub-optimal medication adherence will be offered brief interventions (which can be delivered via a clinician or smartphone app) to enhance treatment engagement and to minimize confounding of lithium non-response with non-adherence.

**Conclusions:**

The paper outlines the rationale, design and methodology of the first study being undertaken by the newly established R-LiNK collaboration and describes how the project may help to refine the clinical response phenotype and could translate into the personalization of lithium treatment.

**Electronic supplementary material:**

The online version of this article (10.1186/s40345-019-0156-x) contains supplementary material, which is available to authorized users.

## Background

Across medical disciplines, there is increasing recognition of the potential utility of personalized or precision diagnostics and therapeutics. Although there are subtle differences in the meaning of terms such as ‘personalized’ and ‘precision’ medicine, the approaches share the same goal, namely to tailor clinical decision-making to each patient by utilizing information about individual phenotypes and genotypes (European Commission 2011). In psychiatry, it is unlikely that personalized diagnostics will be developed for some time as there is uncertainty regarding pathophysiological mechanisms underpinning different mental disorders and no objective laboratory tests are available (Perna et al. 2018; Schumann et al. 2014). However, it may be feasible to help clinicians to customise psychiatric treatment decisions by applying the principles of precision medicine and stratifying patients according to likelihood of response (Scott and Etain 2018). Several multicentre collaborations exist that are fully or partly focused on the identification of biomarkers of psychotropic response, including ongoing studies of antidepressant response (Trivedi et al. 2016); of selected subgroups of patients with bipolar disorders (BD) recruited to randomized controlled trials (RCTs) (Ritter et al. 2016); and of pharmacogenomics of lithium (Li) response (Oedegaard et al. 2016). The Response to Li Network (R-LiNK; https://rlink.eu.com/) is a new European initiative aimed at exploring precision prescribing of Li in BD-I; this project will complement other studies and address some of the gaps in current knowlegde.

In this preliminary communication, we highlight the rationale for developing this prospective multidisciplinary international project, then we provide a brief synopsis of the planned study and comment on the important opportunities and potential difficulties of undertaking this type of multicentre research.

## The Rationale for R-LiNK

The BD diagnostic category comprises a broad range of disorders affecting about 1–3% of the global population. Whilst the diagnosis of BD-II and other bipolar spectrum disorders show poor reliability, BD-I is one of the three most reliable diagnoses in psychiatry and this BD subtype has superior predictive validity for future disease course and outcome (First 2016). In its most severe form, BD-I is associated with considerable morbidity, all-cause premature mortality and a high risk of suicide (Whiteford et al. 2013). Further, a recent study from the USA estimated that the economic burden of BD-I exceeded $200 billion, a cost which Cloutier et al. (2018) suggest illustrates the need to optimize therapeutic strategies. This view concurs with the European College of Neuropsychopharmacology Network Initiative and the European Medicines Agency, which both advocate for international, collaborative research to promote individualized prescribing of established and novel drug treatments in BD.

A critical component of the treatment of BD-I is prevention of relapse/recurrence and reduction in suicidality (Kessing et al. 2018; Tondo and Baldessarini 2018). Clinical and research evidence suggests that Li is one of the most efficacious interventions for targeting these problems and can be beneficial in treating acute BD episodes (Bauer et al. 2018; Vieta et al. 2018). Also, it is the least expensive mood stabilizer available, with a 1-month supply of Li costing only about one dollar (Oedegaard et al. 2016). However, there are difficulties in translating research efficacy of Li into clinical effectiveness in BD-I. For example, response to Li in acute mania does not reliably predict the outcome of prophylactic treatment; many individuals report tolerability issues; and the narrow therapeutic window leads to concerns regarding toxicity (Hayes et al. 2016; Kessing et al. 2018). These problems have all reduced patient and clinician preference for Li as a first line treatment (Zivanovic 2017). Overall, the trial and error approach to prescribing Li for relapse prevention in clinical settings means that its real-world effectiveness is only about half of that reported in research settings and partial- or non-adherence is an important contributor to this treatment failure (Scott and Pope 2002; Howes et al. 2017).

Given the above, it is unsurprising that psychiatrists, patients and their significant others would welcome a more customised approach to prescribing Li prophylaxis in BD-I. Most importantly, there is a need to avoid or considerably curtail the extended trial period (about 18–24 months) that is often required to determine whether there has been a significant reduction in the frequency and severity of relapses/recurrences or a clinically meaningful decrease in illness activity. To date, predictors of Li response employed in clinical practice are contradictory and biomarkers of prophylactic response or tolerability remain tenuous (Kleindienst et al. 2005; Sportiche et al. 2017; Oedegaard et al. 2016; Montlahuc et al. 2019). However, experts involved in clinical and research work with individuals with BD-I have recently reported encouraging preliminary findings regarding response predictors across a range of domains such as omics and neuroimaging (e.g. Silverstone et al. 2005; Hallahan et al. 2011; Bellivier et al. 2013; Roux and Dosseto 2017; Smith et al. 2018). Following discussions, they agreed that several candidate biomarkers of Li response warranted further study. However, the prerequisites for further research were (a) access to large clinically representative samples, and (b) a more systematic ‘integrative science’ approach to examination of clinical response phenotypes (comprising clinical syndrome subtyping and prospective longitudinal monitoring of course of illness during exposure to treatment), and the selected functional, structural, and molecular or metabolic biomarkers (Amare et al. 2017). Sixteen European centres (see Additional file 1: Appendix S1) have formed a multi-national research collaboration focused on the study of the Li response in BD-I. The network aims to provide a basis for precision prescribing of Li and address some of the potential methodological issues highlighted by researchers involved in analogous studies (see Table 1 for examples of reported issues and R-LiNK approach).

**Table 1:** Summary of approaches used by current studies of precision psychiatry compared with the R-LiNK study (see text for details)

Current approach	R-LiNK strategy
Studies of lithium response biomarkers are largely based on secondary analyses of efficacy RCTs, or convenience samples. The lack of clinical representativeness and/or small sample sizes may have contributed to biases in findings in biomarker studies (Carvalho et al. 2016; Hoertel et al. 2013)	To increase the translational potential, the R-LiNK study employs a pragmatic design that reflects clinical reality. Each centre will recruit 20–30 patients who have agreed to initiate a trial of lithium treatment (on the recommendation of their treating clinician). Exclusion criteria are minimized to enhance the generalizability and external validity of study findings
The definition and measurement of lithium response varies between publications. Some studies focus only on ‘good response’ subgroups and compare this group to the rest of the population; others identify several response categories or compare a range of categorical and continuous measures of response. Many studies assess lithium response using cross- sectional retrospective assessments rather than prospective monitoring. Also, only few studies follow guidelines on differentiating non-response from non-adherence (Howes et al. 2017)	R-LiNK will follow participants prospectively for 2 years after initiation of lithium and will systematically assess clinical symptoms, illness activity and medication adherence over time. An independent panel of experts will examine all this longitudinal data to classify the clinical response of each participant according to response categories that have been agreed a priori (with the aim of reducing phenotypic misclassifications) Lithium adherence and risk of sub-optimal adherence will be monitored. A brief intervention may be offered to maintain engagement with treatment
Samples vary in homogeneity or heterogeneity, in the reliability of diagnosis and the range of BD subtypes included Measures of illness activity may vary significantly across studies: some use retrospective clinical reports, others use established observer ratings, others combine observer and objective ratings. The type of ratings selected, and the weightings given to individual symptoms of BD or to illness dimensions in the scales selected may affect the identification of clinical predictor variables or influence the concordance between clinical and biological variables Few biomarker studies include patient-related outcomes (PRO) Many components of the methodology adversely affect the potential signal-to-noise ratio (South et al. 2017)	R-LiNK will recruit individuals with symptoms that meet internationally accepted diagnostic criteria for BD-I and includes methodological strategies that try to increase the ‘signal’ and reduce the ‘noise’ (Scott and Etain 2018) Symptom measures have been selected based on (i) good psychometric, item response theory (IRT) and clinimetric properties; (ii) the weighting given to symptoms that may be particularly sensitive to change early during lithium treatment (e.g. activity, energy and mood); (iii) a balanced assessment of key symptom dimensions We will use electronic self-monitoring of core BD symptoms. This ecological momentary assessment (EMA) approach will include daily ratings of a unique subset of selected symptoms of BD plus additional PRO items that can be used to formulate individualized ratings of personal recovery (which also can be compared with other R-LiNK response categories)
Many studies focus on a single biomarker or select markers from one specific domain (e.g. focusing only on fMRI, omics, etc.). Recent research indicates that prediction may be enhanced by using combinations of factors, rather than trying to identify single or unidimensional biomarkers of outcome or treatment response (Trivedi et al. 2016)	It is unlikely that there is a single biomarker for lithium response (or non-response or tolerability), so R-LiNK employs an integrative science approach to try to identify combinations of clinical, functional, structural, molecular and metabolic biomarkers (called biosignatures) that may be included in a composite prediction tool. In addition, R-LiNK will examine clinical and biological moderators and mediators of lithium response

The development of R-LiNK and the current project are funded via the H2020 funding stream on personalized medicine (https://cordis.europa.eu/project/rcn/212676/factsheet/en). Another benefit of the collaboration is that it provides an infrastructure to support future multidisciplinary, multicentre research on personalized diagnostics and therapeutics for BD. Also, the network will receive input and feedback from a committee of international experts that monitors and advises on the project. From the start, R-LiNK has fostered links to researchers and initiatives involved in similar studies (e.g. Schulze et al. 2010; Oedegaard et al. 2016), which also may facilitate data sharing.

## Synopsis of the R-LiNK cohort study

The inaugural R-LiNK project comprises a prospective cohort study and the protocol follows STROBE guidelines (Strengthening the Reporting of Observational Studies in Epidemiology; von Elm et al. 2007); its two key objectives are:

(i) To improve the early prediction of response to Li by identifying multi-modal biomarkers or biosignatures;
(ii) To implement new and powerful technologies, such as functional omics (genetics, transcriptomics, metabolomics, etc.), to help characterize the early molecular signature of Li in responders and non-responders; also, selected centres will use lithium-7 magnetic resonance imaging (^7^Li-MRI) to characterize the early steady state distribution of Li in the brain (and examine differences associated with responder status).

The project comprises of several work packages (WP), each of which has specific objectives or tasks (see Table 2). For instance, WP4 includes MRI of specific brain structures (e.g. amygdala) and metabolic imaging (using proton magnetic resonance spectroscopy (^1^H-MRS)) undertaken pre- and post-initiation of Li; whilst WP9 focuses on communication, dissemination and exploitation of findings. Most WP warrant separate articles to fully describe the underlying hypotheses and strategies, so this preliminary communication provides a brief overview of the planned study, highlighting selected components.

**Table 2:** Overview of the R-LiNK work packages

Work package	Aims
1	Project administration
2	Set-up, ethical approval, database development
3	Identification of baseline & follow-up assessment of clinical symptoms, neuropsychological and social functioning, illness activity, etc Characterisation of the clinical response phenotype for lithium treatment according to pre-defined outcome measures (categorical/continuous) Assessment & optimization of medication adherence Economic modelling of using a stratified approach to prescribing lithium; Qualitative & quantitative assessment of the digital phenotype Development of a prototype device for salivary lithium measurement
4	Examination of neuro-imaging (MRI and ^1^H-spectroscopy) signature before & after lithium initiation to allow repeated assessment of e.g. architecture of the amygdala, etc
5	Assessment of 7Li-MRI signature (i.e. distribution of lithium in the brain measured 12 weeks after initiation of treatment)
6	Blood sampling to measure omics (e.g. putative transcriptomic, mirnomic, methylomic and proteomic biomarkers) before & after lithium initiation to explore potential molecular signatures
7	Data management infrastructure for e.g. data collection of heterogeneous data (imaging, genetics, clinical) across different institutions & countries; Quality control of data processing; controlled data sharing; etc Data analysis
8	Evaluation of laboratory to bedside transferability of study findings according to e.g. clinical feasibility, technical feasibility, utility of markers when employed alone or in combination (i.e. additivity or redundancy), acceptability & cost effectiveness
9	Communication & dissemination of findings

The study is being undertaken in accordance with the Revised Declaration of Geneva (Parsa-Parsi 2017). Ethical approval was first obtained in France (18.08.02.40026 RiPH 2), followed by approval by ethics committees in other participating countries. Recruitment of study participants commences during 2019, but such a large-scale collaborative venture requires considerable preparatory work (see WP1 and WP2). So, 2018 was dedicated to establishing management structures; developing and translating clinical assessments; establishing inter-rater reliability; harmonization of study protocols between centres (e.g. for MRI); developing procedures for collection and transportation of blood samples; construction of shared database; establishing study monitoring procedures; etc.

A priority for the project is to maximize the clinical representativeness of the cohort by offering study participation to consecutive cases of BD-I that have agreed to commence a trial of Li (WP3). Each centre will recruit approximately 20 patients (with replacement of early dropouts), but the sample size calculation has allowed for 10–20% attrition during follow-up. To increase the translational potential of the study, there are only three eligibility criteria, namely that a potential participant has: (i) a diagnosis of BD-I confirmed according to internationally recognized diagnostic criteria (American Psychiatric Association 2013); (ii) no known reason for exclusion from the research (i.e. the individual is willing and able to give written informed consent; there is no imminent risk of severe self-harm; etc.); and (iii) no contra-indication to taking Li is identified by the treating clinician or during preliminary clinical and physiological screening. As several linked sub-studies are being undertaken, it is important to consider participant burden, so patients can opt to consent to involvement in some but not all sub-studies. Likewise, some centres will focus their research endeavours of the measurement of specific biomarkers, e.g. some but not all centres will perform ^7^Li-MRI or collect actigraphy data, etc.

The R-LiNK centres have been discouraged from changing their approach to Li prescribing and clinical case management will be undertaken separately and independently of the research protocol. Clinicians involved in the day-to-day treatment of patients will make all decisions regarding initiation and prescription of Li, dose titration and continuation or discontinuation of prophylaxis according to individual patient needs.

**Fig. 1:**
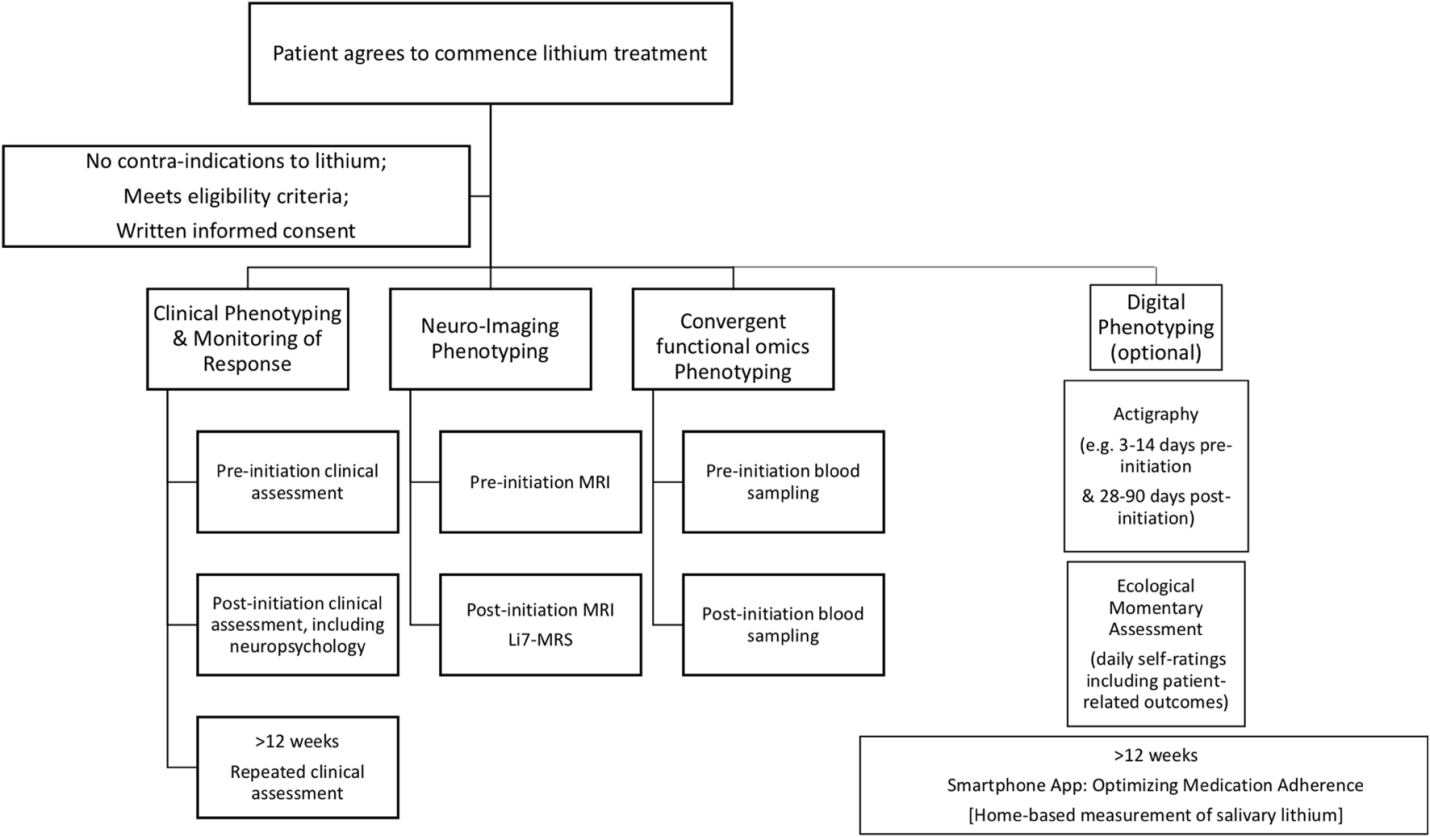
Sequence of assessment of clinical, structural, functional and metabolic markers (see text for details). Pre-initiation refers to the time period between agreement to commence a trial of lithium and actual initiation of medication, and it is expected to average about 2 weeks; most post-initiation measures will be undertaken at approximately week 12 (allowing for titration of lithium dosage & stabilization of plasma levels); neuropsychology assessment will be undertaken in individuals with 4 consecutive weeks of euthymia; actigraphy will ideally be include some days pre-lithium initiation, as some analyses will be feasible with a minimum of 3 days of continuous recording; post-lithium initiation actigraphy may be extended for prolonged periods in patients who consent to this; any program for optimizing adherence will commence after approximately 12 weeks (after stabilization of lithium treatment), when repeated ratings of levels of adherence are available; home-based salivary lithium assessments will only be undertaken in a small subsample of patients who agree to participate in an exploratory pilot study (during the second year of follow-up)

As shown in Fig. 1, the first 12 weeks of the study involve a systematic, detailed baseline clinical assessment (Additional file 2: Appendix S2 provides the list of proposed measures). This occurs alongside the stepwise measurement of structural, functional and molecular or metabolic biomarkers, which is undertaken before and 12 weeks after Li initiation to capture intra-individual biological changes to be tested with the long-term response status. This study phase is followed by prospective longitudinal clinical monitoring of illness activity, medication adherence and response to Li, with reassessments undertaken face to face every 3 months (supplemented by monthly telephone check-ups) for 2 years.

Figure 1 highlights that some assessments and measurements are undertaken only at baseline assessment (e.g. family history of mental disorders) whilst others are repeated before and after initiation of Li (e.g. blood is collected for omics studies to explore any early differential molecular signatures of Li response; MRI is undertaken at baseline and at 12 weeks). In contrast, measurement of Li distribution in the brain is undertaken only once at about 12 weeks); whilst there is an option for actigraphy to be continued over several months.

The R-LiNK project provides an opportunity to extend the search for phenotypes beyond the established architypes (Dawkins 1982). For instance, some centres will explore a putative digital phenotype of Li response in BD-I (a term that describes health data collected from individual monitoring, social media use, and measurement of interactions with technology) (Jain et al. 2015). This sub-study explores the combination of daily self-ratings (e.g. mood, energy, activity) and continuous actigraphic recordings of sleep–wake cycles (Moskowitz and Young 2006; Faurholt-Jepsen et al. 2019; Scott et al. 2017). In keeping with patient and advocacy group requests, the electronic self-monitoring program allows participants to include additional self-selected patient-related outcomes (PRO) (Jonas et al. 2012). Also, a small-scale pilot study will explore self-monitoring and self-management using a prototype device that allows home-based measurement of salivary Li levels.

After all participants have completed the 2-year follow-up period, an international panel of five experts will use a consensus approach to classify each case into the most appropriate response category (see Table 3 reports for selected details from the algorithm and an example of the proposed criteria for classifying Li response). First, the panel will classify cases according to change in illness activity (e.g. evidence of sustained remission or relapse/recurrence that meet accepted criteria (Tohen et al. 2009)) irrespective of degree of exposure to Li across the 2-year prospective follow-up. At the second step, the panel will consider responder category in the context of real-world exposure to Li (dose and duration of Li treatment, serum levels and medication adherence). This important issue is often overlooked in biomarker studies but may contribute to variance in findings or difficulties in interpretation of results (Scott and Pope 2002; Howes et al. 2017). For example, it is possible that a good outcome will be observed in some individuals who are Li non-adherent. This may confound responder analyses, as any biomarkers identified in this subgroup may be predictive of good prognosis in BD-I (independent of adherence), rather than being associated with exposure to Li (Chen et al. 2016; Hou et al. 2016). Hence, in a similar way to the use of intent to treat and per protocol analyses in RCTs, the use of these two strategies will allow the panel to anticipate potential confounders of the clinically observed Li response in the R-LiNK cohort.

**Table 3:** Proposed criteria for classifying lithium response

After all the study participants have completed 2 years of prospective follow-up, clinical response status will be determined by a panel of five experts (who are blinded to biomarkers findings). Using a “best estimate” method (Fennig et al. 1994), the panel will review all available clinical data and provide a consensus classification for each participant according to one of three categories: (i) good responders (GR), (ii) partial responders (PR), (iii) non-responders (NR). If no clinical follow-up data are available, the participant will be categorized as unclassifiable (UC)
Overview of procedure:
(i) The panel will first assess clinical outcomes using internationally agreed consensus definitions of clinical recovery, remission, and partial and full relapse (Tohen et al. 2009) and review illness activity for the 2 years prior to and 2 years after initiation of lithium treatment. Illness activity is a composite measure (e.g. incorporating number of syndromal episodes of bipolar disorder (BD), number of days ill or well during a specified time, etc.). The percentage change in illness activity will be calculated by estimating the difference in the level of illness activity in the 2 years after lithium initiation compared with the 2 years prior to lithium initiation
(ii) Next, the panel will use the evaluation of the clinical outcome to classify the participant in the relevant responder category
For example, for GR:
Classification of response: The expert panel will be asked to classify individuals according to the following steps:
1. All study participants will be classified into the most appropriate responder category irrespective of their exposure to lithium
2. The experts will consider responder status in the context of level of exposure to lithium (e.g. dose, duration of treatment, adherence and serum levels, adjunctive treatments, etc.)
3. Further classifications will examine different conceptualizations of response (e.g. time to remission or relapse, etc.) and other outcomes or putative changes of interest (e.g. sequence of change of symptoms, etc.)
The individual meets criteria for sustained remission, namely they experience euthymia (defined as a score on the Quick Inventory of Depressive Symptoms (qIDS) < 6 and the Bech Rafaelson Mania Scale (BRMS) < 7) for a minimum period ≥ 8 consecutive weeks during the 2-year follow-up period without evidence of relapse into a syndromal episode of BD at any time after achieving sustained euthymia
AND No additional mood stabilizer medication is prescribed after initiation of lithium treatment

Please note it is not possible to reproduce the full document that will be used by R-LiNK, so the Table provides only examples of the relevant guidance and criteria for responder classification

The study includes longitudinal assessment of medication adherence to help identify study participants with sub-optimal adherence and repeated measurement of relevant health beliefs should allow early identification of individuals at future risk of non-adherence (Clatworthy et al. 2009). The aspiration is to develop a brief, evidence-based intervention to enhance medication adherence that can be delivered via a smartphone app or face-to-face (Scott and Tacchi 2002).

### Data management and analysis

The analyses of each biomarker (alone or in combination) will be guided by the approaches used by researchers involved in each WP. In R-LiNK, moderators of treatment effects may include baseline clinical characteristics, or markers derived from tests undertaken early in Li treatment (e.g. neuropsychology). Likewise, potential mediators of Li effects may include e.g. changes in omics or neuroimaging markers (measured prior to commencing Li and at 12 weeks).

Many researchers argue that discovery science strategies are justifiable in precision psychiatry as these approaches are both hypothesis generating and hypothesis-testing (e.g. Mennes 2016; Trivedi et al. 2016). This strategy has been proposed for R-LiNK, which attempts to quantify the predictive value of putative biomarkers and to determine which combinations of markers have additive or interactive effects for identifying an individuals’ likelihood of Li response. A major issue in planning analyses of biosignatures is that such studies collate ‘high dimensional data’ (i.e. repeated measures of multiple putative biomarkers derived from a circumscribed clinical sample of a few hundred participants). An entire work package (WP7) will address harmonization of multimodal biomarker data, the handling of missing data within and across domains, and identification of the most appropriate statistical approaches.

Secondary outcomes will focus on the analysis of different definitions of Li response and will include e.g. analyses of time to response or ‘mirror-image’ approaches can be used to assess changes in illness activity for specified time periods before and after initiation of treatment (such as number of days ill) or intra-individual, day-to-day symptom variability, etc. Another innovation in the R-LiNK study is the inclusion of health economic analyses. Cost-effectiveness has largely been ignored in precision prescribing studies, but it is important to determine whether the introduction of biomarker-driven treatments is economically as well as clinically justifiable (Fugel et al. 2014). Health economics will be examined by constructing simulation models (e.g. mapping current care pathways, and then testing the model after incorporating relevant findings) to assess the impact of stratification on potential costs of treatment for BD-I (of which Li is one element) and changes in outcomes (measured in quality adjusted life years).

## Conclusions

Psychiatry is at a very preliminary stage in its development of personalized approaches and stratification based on disease mechanisms or drug mechanism of action is unfeasible. The current state of our knowledge indicates that the search for phenotypes or ‘responder/non-responder’ subgroups requires a combination of systematic exploration of clinical characteristics (such as treatment-relevant subtypes within the BD-I diagnostic category), prospective longitudinal monitoring of illness activity during treatment, alongside the search for biomarkers derived from several dimensions (e.g. omics, neuro-imaging, actigraphy, etc.). In BD-I, research efforts directed towards the identification of biosignatures for Li response or tolerability are preferable as it is unlikely that a single unidimensional biomarker with robust predictive validity will be found. To date, studies focused solely on single clinical or biological markers have explained only a small degree of variance. Integrative approaches to clinical and multimodal response markers may allow a composite ‘prediction algorithm’ to be developed and tested in new populations. In addition, this project will focus on biological changes induced by Li in each participant (intra-subject design) to limit the risk of spurious findings associated with wide multimodal exploratory strategies. Before the findings can be employed to better inform the clinical decision-making, it will be necessary to determine which biomarkers can be transferred most efficaciously from bench to bedside. This involves feasibility of inclusion in routine clinical practice, patient acceptability, ease of interpretability of tests, etc., and evidence that the costs do not exceed the benefits. Also, we recognise that late lithium response (after 2 years) will not be detected using the current research framework and additional studies will be needed to capture this phenomenon.

The R-LiNK study is an important first step in a process that may ultimately help clinicians and patients to predict the likelihood of response (or non-response or intolerance) to Li prior to or within the first few months of initiating treatment. Already, the R-LINK initiative has delivered a large pan-European multidisciplinary collaborative network with shared protocols for recruitment of clinically representative samples of patients and harmonized procedures for research; this ensures that the network is primed to undertake further studies that can refine eligibility criteria for treatment with other mood stabilizers and could examine precision prescribing of novel drugs in the future.

## Additional files

**Additional file 1: Appendix S1.** Country and location of centres involved in R-LiNK.

**Additional file 2: Appendix S2.** Proposed list of instruments used for baseline clinical assessments and longitudinal monitoring of symptoms, medication adherence and lithium response.

## Data Availability

Core sections of the application are available at https://cordis.europa.eu/project/rcn/. Additional information is available from FB on request.

## Abbreviations

BD: bipolar disorders
BD-I: bipolar I disorder
ConLiGen: The International Consortium on Lithium Genetics
EMA: ecological momentary assessment
^1^H-MRS: proton magnetic resonance spectroscopy
H2020: Horizon 2020
MRI: magnetic resonance imaging
^7^Li-MRI: lithium-7 magnetic resonance imaging
PRO: patient-related outcomes
R-LiNK: Response to Lithium NetworK

## References

[CR1] Amare AT, Schubert K, Baune BT (2017). Pharmacogenomics in the treatment of mood disorders: strategies and opportunities for personalized psychiatry. EPMA J.

[CR2] American Psychiatric Association (2013). Diagnostic and statistical manual of mental disorders.

[CR3] Bauer M, Andreassen O, Geddes J, Kessing L, Lewitzka U, Schulze T, Vieta E (2018). Areas of uncertainties and unmet needs in bipolar disorders: clinical and research perspectives. Lancet Psychiatry.

[CR4] Bellivier F, Geoffroy PA, Scott J, Schurhoff F, Leboyer M, Etain B (2013). Biomarkers of bipolar disorder: specific or shared with schizophrenia?. Front Biosci (Elite Ed).

[CR5] Carvalho AF, Kohler CA, Fernandes BS, Quevedo J, Miskowiak KW, Brunoni AR (2016). Bias in emerging biomarkers for bipolar disorder. Psychol Med.

[CR6] Chen C, Lee C, Chen H, Wu L, Chang J, Liu C, Cheng A (2016). GADL1 variant and medication adherence in predicting response to lithium maintenance treatment in bipolar I disorder. BJPsych Open.

[CR7] Clatworthy J, Bowskill R, Parham R, Rank T, Scott J, Horne R (2009). Understanding medication non-adherence in bipolar disorders using a necessity-concerns framework. J Affect Disord.

[CR8] Cloutier M, Greene M, Guerin A, Touya M, Wu E (2018). The economic burden of bipolar I disorder in the United States in 2015. J Affect Disord.

[CR9] Dawkins R (1982). The extended phenotype: the gene as the unit of selection.

[CR10] European Commission. European perspectives in personalised medicine. – 2011. Luxembourg: Publications Office of the European Union. 2011, p. 36. ISBN 978-92-79-21328-1. 10.2777/75779. http://ec.europa.eu/research/health/pdf/personalised-medicine-conference-report_en.pdf.

[CR11] Faurholt-Jepsen M, Torri E, Cobo J, Yazdanyar D, Palao D, Cardoner N, Andreatta O, Mayora O, Kessing L (2019). Smartphone-based self-monitoring in bipolar disorder: evaluation of usability and feasibility of two systems. Int J Bipolar Disord.

[CR12] Fennig S, Craig T, Lavelle J, Kovasznay B, Bromet EJ (1994). Best-estimate versus structured interview-based diagnosis in first-admission psychosis. Compr Psychiatry.

[CR13] First MB (2016). The importance of developmental field trials in the revision of psychiatric classifications. Lancet Psychiatry.

[CR14] Fugel HJ, Nuijten M, Faulkner E (2014). The application of economics concepts to stratified medicine–use of health economics data to support market access for stratified medicine interventions. J Med Econ.

[CR15] Hallahan B, Newell J, Soares JC, Brambilla P, Strakowski SM, Fleck DE (2011). Structural magnetic resonance imaging in bipolar disorder: an international collaborative mega-analysis of individual adult patient data. Biol Psychiatry.

[CR16] Hayes J, Marston L, Walters K, Geddes J, King M, Osborn D (2016). Lithium vs. valproate vs. olanzapine vs. quetiapine as maintenance monotherapy for bipolar disorder: a population-based UK cohort study using electronic health records. World Psychiatry.

[CR17] Hoertel N, Le Strat Y, Lavaud P, Dubertret C, Limosin F (2013). Generalizability of clinical trial results for bipolar disorder to community samples: findings from the national epidemiologic survey on alcohol and related conditions. J Clin Psychiatry.

[CR18] Hou L, Heilbronner U, Degenhardt F, Adli M, Akiyama K, Akula N (2016). Genetic variants associated with response to lithium treatment in bipolar disorder: a genome-wide association study. Lancet.

[CR19] Howes OD, McCutcheon R, Agid O, de Bartolomeis A, van Beveren NJ, Birnbaum ML (2017). Treatment-resistant schizophrenia: treatment response and resistance in psychosis (TRRIP) working group consensus guidelines on diagnosis and terminology. Am J Psychiatry.

[CR20] Jain S, Powers B, Hawkins J, Brownstein J (2015). The digital phenotype. Nat Biotechnol.

[CR21] Jonas D, Mansfield A, Curtis P, Gilmore J, Watson L, Brode S (2012). Identifying priorities for patient-centered outcomes research for serious mental illness. Psychiatr Serv.

[CR22] Kessing L, Bauer M, Nolen W, Severus E, Goodwin G, Geddes J (2018). Effectiveness of maintenance therapy of lithium vs other mood stabilizers in monotherapy and in combinations: a systematic review of evidence from observational studies. Bipolar Disord.

[CR23] Kleindienst N, Engel R, Greil W (2005). Which clinical factors predict response to prophylactic lithium? A systematic review for bipolar disorders. Bipolar Disord.

[CR24] Mennes M (2016). Commentary: leveraging discovery science to advance child and adolescent psychiatric research–a commentary on Zhao and Castellanos 2016. J Child Psychol Psychiatry.

[CR25] Montlahuc C, Curis E, Grillault Laroche D, Bagoe G, Etain B, Bellivier F, Chevret S (2019). Response to lithium in patients with bipolar disorder: what are psychiatrists’ experiences and practices compared to literature review?. Pharmacopsychiatry.

[CR26] Moskowitz DS, Young SN (2006). Ecological momentary assessment: what it is and why it is a method of the future in clinical psychopharmacology. J Psychiatry Neurosci.

[CR27] Oedegaard KJ, Alda M, Anand A, Andreassen OA, Balaraman Y, Berrettini WH (2016). The pharmacogenomics of bipolar disorder study (PGBD): identification of genes for lithium response in a prospective sample. BMC Psychiatry.

[CR28] Parsa-Parsi RW (2017). The revised declaration of Geneva: a modern-day physician’s pledge. JAMA.

[CR29] Perna G, Grassi M, Caldirola D, Nemeroff CB (2018). The revolution of personalized psychiatry: will technology make it happen sooner?. Psychol Med.

[CR48] Ritter PS, Bermpohl F, Gruber O, Hautzinger M, Jansen A, Juckel G, Kircher T, Lambert M, Mulert C, Pfennig A, Reif A, Rienhoff O, Schulze TG, Severus E, Stamm T, Bauer M (2016). Aims and structure of the German Research Consortium BipoLife for the study of bipolar disorder. Int J Bipolar Disord.

[CR30] Roux M, Dosseto A (2017). From direct to indirect lithium targets: a comprehensive review of omics data. Metallomics.

[CR31] Schulze TG, Alda M, Adli M, Akula N, Ardau R, Bui ET (2010). The International Consortium on Lithium Genetics (ConLiGen): an initiative by the NIMH and IGSLI to study the genetic basis of response to lithium. Neuropsychobiology.

[CR32] Schumann G, Binder EB, Holte A, de Kloet ER, Oedegaard KJ, Robbins TW (2014). Stratified medicine for mental disorders. Eur Neuropsychopharmacol.

[CR33] Scott J, Etain B (2018). Bellivier F Can an integrated science approach to precision medicine research improve lithium treatment in bipolar disorders?. Front Psychiatry.

[CR34] Scott J, Pope M (2002). Self-reported adherence to treatment with mood stabilizers, plasma levels, and psychiatric hospitalization. Am J Psychiatry.

[CR35] Scott J, Tacchi MJ (2002). A pilot study of concordance therapy for individuals with bipolar disorders who are non-adherent with lithium prophylaxis. Bipolar Disord.

[CR36] Scott J, Murray G, Henry C, Morken G, Scott E, Angst J, Merikangas KR, Hickie IB (2017). Activation in bipolar disorders: a systematic review. JAMA Psychiatry.

[CR37] Silverstone PH, Bell EC, Wilson MC, Dave S, Wilman AH (2005). Lithium alters brain activation in bipolar disorder in a task- and state-dependent manner: an fMRI study. Ann Gen Psychiatry.

[CR38] Smith FE, Thelwall PE, Necus J, Flowers CJ, Blamire AM, Cousins DA (2018). 3D (7)Li magnetic resonance imaging of brain lithium distribution in bipolar disorder. Mol Psychiatry.

[CR39] South C, Rush AJ, Carmody TJ, Jha MK, Trivedi MH (2017). Accurately identifying patients who are excellent candidates or unsuitable for a medication: a novel approach. Neuropsychiatr Dis Treat..

[CR40] Sportiche S, Geoffroy PA, Brichant-Petitjean C, Gard S, Khan JP, Azorin JM, Henry C, Leboyer M, Etain B, Scott J, Bellivier F (2017). Clinical factors associated with lithium response in bipolar disorders. Aust N Z J Psychiatry.

[CR41] Tohen M, Frank E, Bowden CL (2009). The International Society for Bipolar Disorders (ISBD) task force report on the nomenclature of course and outcome in bipolar disorders. Bipolar Disord.

[CR42] Tondo L, Baldessarini RJ (2018). Antisuicidal effects in mood disorders: are they unique to lithium?. Pharmacopsychiatry.

[CR43] Trivedi MH, McGrath PJ, Fava M, Parsey RV, Kurian BT, Phillips ML (2016). Establishing moderators and biosignatures of antidepressant response in clinical care (EMBARC): rationale and design. J Psychiatr Res.

[CR44] Vieta E, Berk M, Schulze TG, Carvalho AF, Suppes T, Calabrese JR, Gao K, Miskowiak KW, Grande I (2018). Bipolar disorders. Nat Rev Dis Primers.

[CR45] von Elm E, Altman D, Egger M, Pocock S, Gotzsche P, Vandenbroucke J, STROBE Initiative (2007). The strengthening the reporting of observational studies in epidemiology (STROBE) statement: guidelines for reporting observational studies. Lancet.

[CR46] Whiteford HA, Degenhardt L, Rehm J, Baxter AJ, Ferrari AJ, Erskine HE (2013). Global burden of disease attributable to mental and substance use disorders: findings from the Global Burden of Disease Study 2010. Lancet.

[CR47] Zivanovic O (2017). Lithium: a classic drug-frequently discussed, but, sadly, seldom prescribed!. Aust N Z J Psychiatry.

