# Supplementary material for: Prospective cohort study of early biosignatures of response to lithium in bipolar-I-disorders: overview of the H2020-funded R-LiNK initiative
Source: Int J Bipolar Disord. 2019 Sep 25;7:20. doi: 10.1186/s40345-019-0156-x (PMC6760458; doi:10.1186/s40345-019-0156-x)
Supplement: Supplementary file 1 — Additional file 1: Appendix S1. Country and location of centres involved in R-LiNK. [file 40345_2019_156_MOESM1_ESM.docx]

Appendix S1: Country and location of centres involved in R-LiNK

| COUNTRY | CENTRES |
| --- | --- |
| Denmark | Copenhagen |
|  |  |
| France | Paris |
|  | Colombes |
|  | Besancon |
|  |  |
| Germany | Dresden |
|  | Mainz-Frankfurt |
|  | Munich |
|  |  |
| Italy | Milan |
|  | Brescia |
|  |  |
| Norway | Oslo |
|  | Bergen |
|  |  |
| Spain | Barcelona  (2 centres) |
|  |  |
| Sweden | Gothenburg |
|  |  |
| United Kingdom | London |
|  | Newcastle |
