# Supplementary material for: Prospective cohort study of early biosignatures of response to lithium in bipolar-I-disorders: overview of the H2020-funded R-LiNK initiative
Source: Int J Bipolar Disord. 2019 Sep 25;7:20. doi: 10.1186/s40345-019-0156-x (PMC6760458; doi:10.1186/s40345-019-0156-x)
Supplement: Supplementary file 2 — Additional file 2: Appendix S2. Proposed list of instruments used for baseline clinical assessments and longitudinal monitoring of symptoms, medication adherence and lithium response. [file 40345_2019_156_MOESM2_ESM.docx]

Appendix 2: Proposed list of instruments used for baseline clinical assessments and longitudinal monitoring of symptoms, medication adherence and lithium response

| **Measure** | **Baseline** | **Follow-up** |
| --- | --- | --- |
| Demographic questions | X |  |
| Diagnostic assessment (Mood section of the SCID-5)  (to confirm BD-I diagnosis; exclude schizoaffective disorder, etc) | X |  |
| Standardized Assessment of Personality: Abbreviated Scale (SAPAS) | X |  |
| Clinical and treatment history information (SCID-5) | X |  |
| Family history of mental illness | X |  |
| Childhood Trauma Questionnaire (CTQ) | X |  |
| Personal history of psychiatric comorbidities | X | X |
| Physical health & illness | X | X |
| Brief Psychiatric Rating Scale (BPRS) | X | X |
| Generalised Anxiety Disorder Assessment (GAD-7) | X | X |
| Alcohol, Smoking and Substance Involvement Screening Test (WHO-ASSIST) | X | X |
| Current medication inventory (including name, class, dose & duration of treatment) | X | X |
| Columbia-Suicide Severity Rating Scale | X | X |
| Quick Inventory of Depressive Symptoms (qIDS)* | X | x |
| Bech-Rafaelson Mania Scale (BRMS)* | X | x |
| Clinical Global Impression – bipolar disorder version (CGI-BD) | X | X |
| WHO 2-item Disability Assessment Schedule (WHODAS) | X | X |
| Screen for Cognitive Impairment (SCIP) | X | X |
| Internal States Scale (ISS) | X | X |
| Patient Rated Inventory of Side Effects (PRISE) | X | X |
| Tablet Routine Questionnaire (TRQ) | X | X |
| Medication Adherence Rating Scale (MARS) | X | X |
| Beliefs about Medication Questionnaire (BMQ) | X | X |
| Client Service Receipt Inventory (CSRI) | X | X |
| EuroQol (EQ-5D) | X | X |
| Longitudinal Interval Follow-up Evaluation (LIFE-II) |  | X |
| Retrospective Assessment of Lithium Response (Alda Scale) |  | X |

*qIDS and BRMS ratings during follow-up are included in the LIFE-II
